# Non-intubated Thoracoscopic Surgery to Minimize Contamination From Airway Secretions During the COVID-19 Pandemic

**DOI:** 10.3389/fsurg.2022.818824

**Published:** 2022-02-18

**Authors:** Man-Ling Wang, Ming-Hui Hung, Hsao-Hsun Hsu, Ya-Jung Cheng, Jin-Shing Chen

**Affiliations:** ^1^Department of Anesthesiology, National Taiwan University Hospital and National Taiwan University College of Medicine, Taipei, Taiwan; ^2^Graduate Institute of Clinical Medicine, National Taiwan University College of Medicine, Taipei, Taiwan; ^3^Division of Thoracic Surgery, Department of Surgery, National Taiwan University Hospital and National Taiwan University College of Medicine, Taipei, Taiwan; ^4^Department of Anesthesiology, National Taiwan University Cancer Center, Taipei, Taiwan; ^5^Department of Surgical Oncology, National Taiwan University Cancer Center, Taipei, Taiwan

**Keywords:** thoracic surgery, video-assisted thoracic surgery, non-intubated thoracic surgery, coronavirus disease (COVID-19), personal protective equipment (PPE)

## Abstract

**Background:**

General anesthesia and tracheal intubation potentially pose a high risk to health care workers (HCWs) managing surgical patients during the coronavirus disease 2019 (COVID-19) pandemic. Non-intubated anesthesia is a rational way of managing patients undergoing thoracoscopic surgery that avoids tracheal intubation and minimizes the aerosols generated during airway instrumentation. The purpose of this study was to determine whether non-intubated anesthesia in combination with a face mask is safe and feasible in patients undergoing thoracoscopic surgery.

**Methods:**

A total of 18 patients who underwent non-intubated thoracoscopic surgery with a face mask during the perioperative period between March 9, 2020 and April 6, 2020 were included. The main outcomes were anesthetic management and postoperative results.

**Results:**

The 18 patients had a mean age of 64 years and a body mass index of 22.9 kg/m^2^. All patients wore a mask during induction of anesthesia and throughout surgery. Three patients underwent lobectomy, four segmentectomy, ten wedge resection, and one underwent anterior mediastinal tumor resection. No patient developed cough or vomiting during the perioperative period. All patients were transferred to the postoperative recovery unit within 15 min of the end of surgery (average 7.2 min). No patient required conversion to tracheal intubation or conversion to thoracotomy.

**Conclusion:**

Non-intubated anesthesia with a mask was safe and feasible in patients undergoing thoracoscopic surgery. Avoidance of intubated general anesthesia and use of a lung separation device may reduce the risk to HCWs of contamination by airway secretions, thereby conserving personal protective equipment, especially during the COVID-19 pandemic.

## Introduction

There has been a dramatic increase in numbers of cases of coronavirus disease 2019 (COVID-19) in Europe, the US, and other countries worldwide, and the COVID-19 outbreak has been characterized as a pandemic since mid-March 2020. The rapid increase in medical needs in response to this pandemic has paralyzed health care systems, resulting in shortages of key medical supplies, health care workers (HCWs), critical care resources, and even personal protective equipment (PPE) (1, 2). During this crisis, N95 respirator masks are reserved for aerosol-generating procedures (AGPs) in patients with confirmed or suspected COVID-19 infection. However, asymptomatic carriers can transmit infection during a variable incubation period (3, 4). The level of protection may have to be upgraded even when managing unsuspected patients during a pandemic (5).

Tracheal intubation and associated AGPs, including endotracheal tube suctioning and fiberoptic bronchoscopy, have been identified as the most important risk factors for nosocomial transmission of airborne infections to HCWs (6). General anesthesia and tracheal intubation may pose a higher risk to HCWs when managing surgical patients during the COVID-19 pandemic. Therefore, regional anesthesia is preferable to general anesthesia whenever possible to avoid airway manipulations (7, 8). During surgery, the patient should also wear a surgical mask to reduce the risk of droplet spread (9).

General anesthesia with tracheal intubation and one-lung ventilation are traditionally considered mandatory in thoracic surgery, especially video-assisted thoracoscopic surgery (10). Airway management, including tracheal intubation and extubation, increases the risk of exposure to respiratory droplets for anesthesia providers (11–13). Use of lung separation devices, which typically involve bronchoscopic examination, is also associated with transmission of highly contagious diseases to personnel in the operating room (14, 15). Airway management in patients undergoing thoracic surgery becomes even more challenging at times when there is a risk of acquiring severe acute respiratory syndrome (SARS), Ebola, Middle East respiratory syndrome (MERS), or COVID-19.

Thoracoscopic surgery without intubation has been proposed as a way of reducing the adverse effects associated with tracheal intubation and general anesthesia (16–19). Non-intubated thoracoscopic surgery includes regional anesthesia, targeted sedation, and maintenance of spontaneous breathing in the patient throughout the procedure. Non-intubation is a rational way of minimizing the aerosols generated during airway instrumentation in patients undergoing thoracic surgery and the associated risk to HCWs. In this study, we retrospectively analyzed the safety and feasibility of performing non-intubated thoracic surgery in combination with a face mask.

## Materials and Methods

### Patient Selection

This retrospective study was approved by the Research Ethics Committee of National Taiwan University Hospital (202004023RIN). All patients gave their consent to a non-intubated technique after an explanation of the anesthesia and surgical procedures before undergoing surgery. We started our non-intubated thoracoscopic surgery program for diagnosis and treatment of lung cancer in August 2009 (16). Since then, we have modified our patient selection criteria to avoid the need for conversion to tracheal intubation using the experience accumulated from thousands of cases over the past 10 years (18). The thoracic surgery team, including both surgeons and anesthesiologists, now uses a non-intubated approach in patients undergoing scheduled thoracoscopic surgery whenever possible. Patients are considered candidates for non-intubated thoracoscopic surgery if they have a peripheral lung tumor smaller than 6 cm in diameter without evidence of involvement of the chest wall, diaphragm, main bronchus, or major vessels on computed tomography (CT) of the chest. Patients are considered inappropriate for a non-intubated procedure if they have a body mass index > 30 kg/m^2^, an American Society of Anesthesiologists score > 3, coagulopathy, sleep apnea, a potential or confirmed difficult airway, or abnormal spine anatomy.

### Anesthetic Management

**Figure 1:**
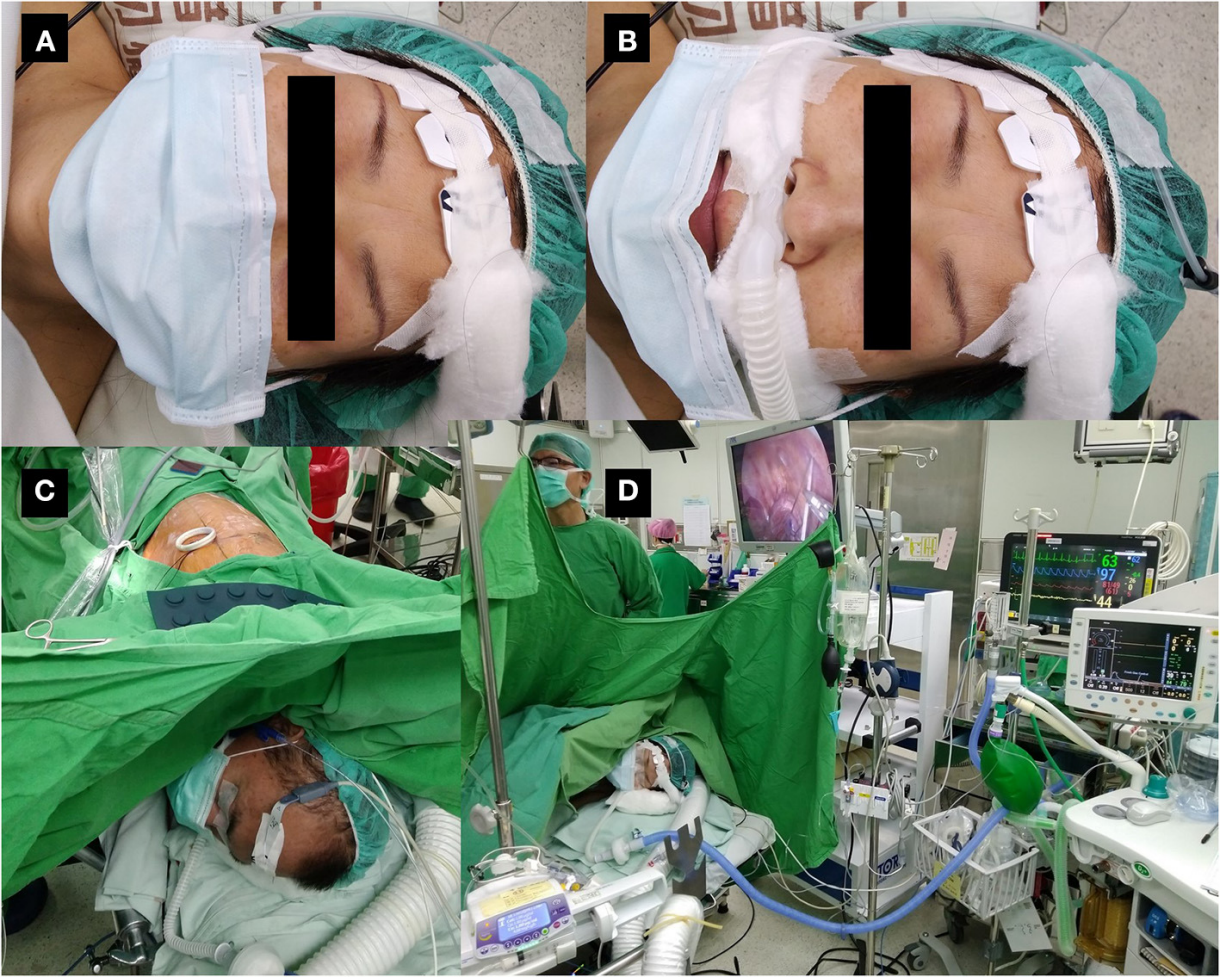
Patient wearing a surgical mask during non-intubated thoracoscopic surgery. **(A)** Non-intubated patient wearing a surgical mask at the time of induction of anesthesia. **(B)** The same patient with the mask pulled down to demonstrate monitoring of exhaled carbon dioxide and high-flow nasal cannula under the mask. The mask will be re-covered before induction of anesthesia. **(C)** Patient undergoing non-intubated thoracoscopic surgery with a surgical mask. **(D)** Operation settings during non-intubated thoracoscopic surgery.

All patients are fitted with a surgical mask before entering the operating room. The anesthesia provider ensures that the mask covers the nasal high-flow cannula from the time of induction of anesthesia onwards (Figure 1). The patient is then preoxygenated with nasal high-flow oxygen at an initial flow rate of 20 l/min (20). Pulse oximetry, electrocardiography, arterial blood pressure, and frontal bispectral index (BIS Quatro; Aspect Medical Systems, Norwood, MA, USA) are monitored continuously. A detector is placed in front of the nose or mouth to monitor end-tidal carbon dioxide. The patients are premedicated with 25–50 mcg of intravenous fentanyl, and then sedated with an intravenous target-controlled infusion of propofol (Injectomat® TIVA Agilia; Fresenius Kabi GmbH, Graz, Austria), as described elsewhere (17). The level of sedation is maintained at a BIS value of 40–60.

The patient is placed in a lateral decubitus position for thoracoscopic surgery. Fentanyl is injected incrementally to maintain a respiratory rate of < 20 breaths/min. The oxygen flow is temporarily suspended immediately before iatrogenic pneumothorax and resumed after confirmation of one-lung ventilation by the surgeon. Oxygen flow is limited to 20 l/min to maintain oxygen saturation above 90% throughout the procedure.

### Iatrogenic Pneumothorax and One-Lung Ventilation

**Figure 2:**
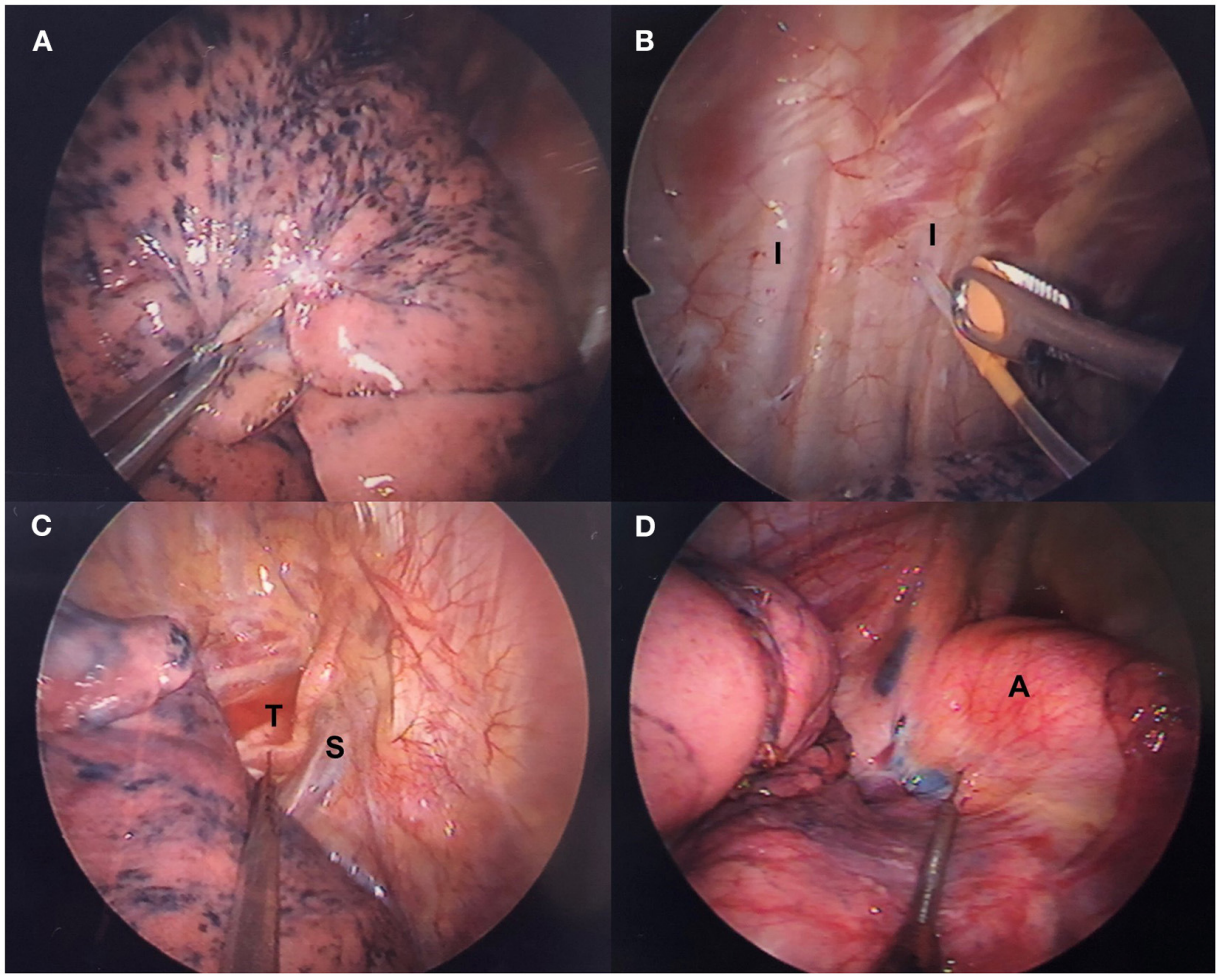
Thoracoscopic intercostal nerve blocks and vagal nerve block. **(A)** Lung collapses gradually after iatrogenic pneumothorax. **(B)** Intercostal nerve blocks are administered with a 25-G top-winged infusion needle under thoracoscopic guidance. **(C)** Right-sided and **(D)** left-sided intrathoracic vagal nerve block in accordance with the operative side. A, aortic arch; I, intercostal nerve; S, superior vena cava; T, trachea.

The first incision is made after local infiltration anesthesia with 2% lidocaine at the fifth intercostal space. An iatrogenic pneumothorax is created after dissecting into the thoracic cavity, and the lung collapses gradually while the patient continues to breath spontaneously (Figure 2A). The oxygen flow is temporarily suspended to facilitate collapse of the lung. The wound is then retracted by a wound protector (Applied Medical, Santa Margarita, CA, USA) to maintain easy access to the working space and protect it.

### Intrathoracic Intercostal Nerve Block

Intrathoracic nerve block has mostly replaced insertion of an epidural catheter for thoracoscopic surgery at our center. Under thoracoscopic guidance, a 25-G top-winged infusion needle is used to achieve analgesia of the chest wall with intrathoracic intercostal nerve blocks by infiltrating 0.5% bupivacaine (1.5 ml for each intercostal space) from the third to eighth intercostal nerve under the parietal pleura 2 cm lateral to the sympathetic chain (Figure 2B). For procedures that take longer than 2 h, the intercostal nerve block is repeated before wound closure.

### Intrathoracic Vagal Block

Intrathoracic vagal block is performed routinely at our center to eliminate the cough reflex, which is induced by manipulation of the lung without muscle relaxation. Under thoracoscopic guidance, intrathoracic vagal nerve block is achieved by infiltration of 3 ml of 0.5% bupivacaine. For a right-sided procedure, the vagal nerve can be easily identified and blocked at the level of the lower trachea (Figure 2C). For a left-sided procedure, vagal block is produced at the level of the aortopulmonary window (Figure 2D).

### Surgical Technique

After collapse of the lung, incomplete fissures, pulmonary vessels, and the bronchi are divided using an endoscopic stapling device. The resected lung parenchyma is removed in an organ retrieval bag. For lobectomy and segmentectomy, hilar dissection is accomplished by electrocautery or use of a harmonic scalpel. For indeterminate nodules, the specimen is sent for intraoperative frozen section biopsy. When the result indicates primary lung cancer, additional resection is performed to ensure an adequate safety margin and lymphadenectomy is performed for complete tumor staging. After staging the mediastinal lymph node dissection, a 28-French chest tube is inserted into the posterior aspect of the lung. Rib spreading, rib cutting, and use of a retractor are avoided, except when conversion to thoracotomy is required.

At the end of the procedure, the collapsed lung is recruited with a high flow of oxygen at 70 l/min to check for air leak by thoracoscopic observation. Infusion of propofol is then stopped. After insertion of a chest tube and closure of the wound, the patient is woken and asked to breathe deeply and hold their breath to expand the previously collapsed lung. The mask covers the patient's nose and mouth throughout the procedure.

### Postoperative Care and Recovery

Patients can resume water and food intake within 2 h of the operation. Oral analgesics are started soon after resumption of oral intake. Intravenous morphine is administered as a recue in the intensive care unit, and nalbuphine in the surgical ward. A plain chest film is obtained immediately after surgery or on the following morning. The chest tube is removed if no air leak is detected and fluid drainage is < 200 ml per day.

### Data Collection and Statistical Analysis

The main outcome variables investigated in this series of patients were the perioperative cough, conversion, mortality, and complication rates. Secondary outcomes included the anesthesia and operating times, rates of vomiting and sore throat, length of postoperative hospital stay, and duration of chest drainage. Information on patient demographics, length of hospital stay, duration of chest drainage, complication rates, and surgical results was collected from the institutional database, anesthesia records, operating notes, and medical and nursing records. Numerical data are shown as the mean, standard deviation, and range. Categorical data are presented as the number (percentage).

## Results

Between March 9, 2020 and April 6, 2020, non-intubated thoracoscopic surgery with a surgical mask was performed in 18 patients with a mean age of 64 years and a body mass index of 22.2 kg/m^2^. The patient characteristics are summarized in Table 1. Most patients (67%, 12/18) were women and three were ex-smokers. One patient had anterior mediastinal tumor, three were confirmed to have lung cancer before surgery, and fourteen had indeterminate lung nodules (six of whom underwent CT-guided dye localization just before surgery).

**Table 1:** Patient demographic and clinical characteristics.

**Variable**	***n* (%)**
^*^Age, years	64.2 ± 10.1 (34.8–80.2, 64.1)
**Female sex**	12 (67)
^*^Height, cm	160.9 ± 8.8 (151–175, 160.2)
^*^Body mass index, kg/m ^**2**^	22.9 ± 2.0 (19.4–26.7, 22.9)
**Comorbidity**
Hypertension	7 (39)
Heart disease	2 (11)
Diabetes mellitus	1 (6)
Extrapulmonary cancer	3 (17)
**Smoking status**
Ever	1 (6)
^*^PFT: FEV_**1**_ **, %**	108.7 ± 22.4 (78.4–172.8, 99.5)
**ASA classification**
2	10 (56)
3	8 (44)

*ASA, American Society of Anesthesiologists; FEV_1_, forced expiratory volume in 1 second; PFT, pulmonary function test*.

* *Presented as mean ± standard deviation (range, median)*.

Intravenous fentanyl was administered as premedication at a dose of 50 mcg in 3 patients and 25 mcg in the remainder. Total fentanyl consumption ranged from 25 to 100 mcg. Five patients did not require an incremental dose. Intraoperative frozen section examinations were performed in 14 of the 18 patients; 9 were confirmed to have primary lung cancer. Three patients underwent lobectomy with lymphadenectomy, 4 underwent segmentectomy with lymphadenectomy, 6 underwent wedge resection with lymphadenectomy, 4 underwent wedge resection without lymphadenectomy, and 1 underwent anterior mediastinal tumor resection (Table 2). There were no intraoperative complications. No patient required conversion to intubated general anesthesia or to thoracotomy. No blood transfusions were required.

**Table 2:** Anesthesia and intraoperative details.

**Variable**	***n* (%)**
**CT-guided dye localization**	6 (33)
**Intraoperative frozen section examination**	14 (78)
**Laterality**
Right	14 (78)
Left	4 (22)
**Operative lobe**
Right upper	9 (50)
Right middle	3 (17)
Right lower	2 (11)
Left upper	2 (11)
Left lower	2 (11)
**Operative method**
Lobectomy with lymphadenectomy	3 (17)
Segmentectomy with lymphadenectomy	4 (22)
Wedge resection with lymphadenectomy	6 (33)
Wedge resection	4 (22)
Anterior mediastinal tumor resection	1 (6)
^*^Operating time, min	109.2 ± 35.0 (62–193, 100)
^*^Emergence duration, min	7.2 ± 3.1 (1–12, 6.5)
**Conversion to thoracotomy**	0
**Conversion to intubated general anesthesia**	0

*CT, computed tomography*.

* *Presented as mean ± standard deviation (range, median)*.

No patient developed cough or vomiting and all patients wore a mask from the time of induction of anesthesia and throughout surgery. At the end of surgery, intravenous morphine was administered at a dose of 3 mg in 7 patients and 2 mg in 7 patients; the remaining 4 patients did not require opioids. All patients resumed oral intake soon after surgery and were managed with oral analgesics (mainly acetaminophen and a non-steroidal anti-inflammatory drugs with or without tramadol). On the day after surgery, one patient required 3 mg of intravenous morphine, one required 10 mg, and two required 4 mg of intravenous nalbuphine as rescue medication. No patient required intravenous opioid on the first postoperative day and in the following days.

The mean time from induction of anesthesia to departure of the patient from the operating room was 109.2 min. The mean operating time was 123.4 min for anatomical resections, including lobectomy and segmentectomy (both with lymphadenectomy), and 97 min for wedge resection. Importantly, all patients were transferred to the postoperative recovery unit within 15 min of completion of surgery, and the mean time until emergence was 7.2 min. All patients continued to wear the surgical mask during their stay in the postoperative care unit.

The postoperative results are summarized in Table 3. One 80-year-old patient remained in the intensive care unit for a day. This patient had clinical stage IIB adenocarcinoma of the left upper lobe and underwent non-intubated thoracoscopic left upper lobe S1–S3 segmentectomy with lymphadenectomy. His postoperative course was uneventful and the chest tube was removed on postoperative day 4. In the other patients, the average duration of chest tube drainage was 2.3 days. No patient complained of sore throat after surgery. One patient developed subcutaneous emphysema after surgery but there were no cases of a leak for more than 3 days. Four patients had postoperative vomiting.

**Table 3:** Postoperative results.

**Variable**	***n* (%)**
**Anesthetic and operative side effects**
Headache	0
Sore throat	0
Urinary retention	0
Vomiting	4 (22)
^*^NRS pain score on the first postoperative day	1.6 ± 1.0 (1–3; 2)
**Operative complications**
Air leak for more than 3 days	0
Blood transfusion	0
Subcutaneous emphysema	1 (6)
**Admission to intensive care unit**	1 (6)
^*^Duration of chest tube drainage, days	2.3 ± 0.6 (2–4, 2)
^*^Postoperative hospital stay, days	3.7 ± 1.0 (3–6, 3)

*NRS, Numeric Rating Scale*.

* *Presented as mean ± standard deviation (range, median)*.

The final pathology reports are summarized in Table 4. Twelve the 18 patients had primary lung cancer, 1 had bronchiolitis obliterans organizing pneumonia, 2 had granulomatous inflammation with caseating necrosis, 1 had granulomatous inflammation, 1 had organizing pneumonia, and 1 had thymic cyst. Seven of the 12 patients with primary lung cancer had clinical stage IA disease, 3 had stage IB, 1 had stage IIB, and 1 had stage IVA.

**Table 4:** Pathological diagnosis.

**Variable**	***n* (%)**
**Primary lung cancer**	12 (67)
Stage IA	7
Stage IB	3
Stage IIB	1
Stage IVA	1
**Bronchiolitis obliterans organizing pneumonia**	1 (6)
**Granulomatous inflammation with caseating necrosis**	2 (11)
**Granulomatous inflammation**	1 (6)
**Organizing pneumonia**	1 (6)
**Thymic cyst**	1 (6)

## Discussion

Our results indicate that non-intubated anesthetic management in combination with a surgical mask is safe and effective in patients scheduled for thoracoscopic surgery. During surgery, the patients in this series maintained spontaneous breathing and airway instrumentation was avoided. The coverage of the surgical mask did not interfere one-lung ventilation and lung recruitment. The surgical field was satisfactory. There were no cases of cough or vomiting during the procedure and all patients emerged from anesthesia rapidly. There were no reports of sore throat after surgery and there was no prolonged air leak. Non-intubated anesthesia with a mask was safe and feasible for management of patients undergoing thoracic surgery and had the potential benefit of decreasing the risk of contamination by airway secretions.

Non-intubated thoracoscopic surgery obviates the need for tracheal intubation and extubation, which usually induce cough and spread of secretions. It also eliminates the need for bronchoscopic examination to check the position of a lung separation device, use of which is discouraged during the COVID-19 pandemic (21). All of these procedures carry a high risk of transmission of a contagious disease to surgical HCWs and are best avoided (13, 22). The risks associated with these procedures were avoided using the non-intubated technique described here. We also believe that our non-intubated technique has helped to conserve our PPE supplies at a time of global shortage.

Use of masks has been advocated to protect against respiratory infection during a pandemic (23–25). In patients with known or suspected COVID-19, the highest level of PPE is recommended for HCWs when providing AGPs. Routine use of N95 respirators may not be feasible or possible when PPE is in short supply. Although wearing a mask is not recommended for individuals without respiratory symptoms, we suggest that all patients wear a mask if they are admitted to hospital. Physical distancing is difficult in a hospital, and nosocomial transmission of COVID-19 is catastrophic. A surgical mask may reduce the risk of transmission of infection during surgery in which neither intubated general anesthesia nor an N95 respirator is required.

Most patients in this series were women (67%) and the mean body mass index was 22.9 kg/m^2^. Our team has performed non-intubated thoracoscopic surgery in thousands of patients, and nasal high-flow oxygen is used routinely to improve arterial oxygenation (18, 20). One of the major reasons for conversion to intubation is an unfavorable breathing pattern, which is associated with obesity and anatomical resections. The non-intubated technique has been evaluated in the management of solitary lung nodules, pleural disease, pneumothorax, lung volume reduction, and lung cancer surgery (16, 18, 26–32). Anatomical resection was performed in 7 (39%) of the 12 patients in this series who underwent primary lung cancer surgery, and lymphadenectomy involving hilar dissection was performed in all cases. Use of an intrathoracic vagal nerve block inhibited the cough reflex, which enabled extensive manipulation of the lung, and eliminated cough-associated aerosol generation and the risk of contamination by secretions, which is common during airway management.

Another advantage of non-intubated anesthesia is that the rate of vomiting is lower than that with intubated general anesthesia, 16 possibly because of use of total intravenous anesthesia and intercostal nerve block with an opioid-sparing effect (33). In our series, all of the 4 patients who developed vomiting after surgery were women and non-smokers. Three of them were prescribed oral tramadol, and one was associated with intravenous nalbuphine. Vomiting ceased in one of these patients after discontinuation of tramadol. General anesthesia, female sex, and non-smoking status have been identified as risk factors for postoperative nausea and vomiting (34). Opioids may be best reserved as rescue medication rather than used as routine analgesia after surgery in patients who are at high risk for postoperative nausea and vomiting.

In the 14 patients who had a preoperative diagnosis of indeterminate lung nodules, 9 were confirmed to have primary lung cancer. With low-dose CT screening, the diagnosis of early-lung cancer became possible (35, 36). When it came to small, deeply located, or ground-glass characteristic lesions, preoperative CT-guided localization was effective to identify the tumors and estimate the resection margins (37, 38). In this cohort, 4 patients had sub-centimeter lung cancer. Of them, two underwent segmentectomy with lymphadenectomy, and the other two underwent wedge resection with lymphadenectomy. Both surgical methods yield satisfactory postoperative results (39).

Although the patients wear a surgical mask, the gas exchange generally follow the effects of using a nasal high-flow oxygen cannula in non-intubated thoracic surgery (20). Compared with traditional intubated general anesthesia with lung isolation, hypercapnia during non-intubated thoracoscopic surgery is inevitable but clinically well tolerated (40). Permissive hypercapnia is also considered to be protective to improve ventilation/perfusion match and modulate inflammatory response (41, 42). We did not use recruitment maneuver in those patients, however, assisted ventilation should reserve most patients with hypercapnia during non-intubated thoracic surgery (43). Moreover, spontaneous and negative-pressure breathing is essential for operative lung to collapse. Without lung isolation, the recruitment maneuver will lead to inflation of both lungs. We usually preserve recruitment maneuver to check for air leakage through thoracoscopic observation at the end of the procedure when we use an oxygen mask (20).

This study has several limitations. First, it had a retrospective design and patients were selected on the basis of a protocol that has been established for years. Second, the data presented were derived from a single institution with expertise in non-intubated thoracoscopic surgery. Therefore, caution is needed when generalizing the observed outcomes to broader clinical practice. Third, the report is based on a case series with no control group for comparison. Researchers are encouraged to conduct further clinical studies to provide solid evidence that wearing a surgical mask during non-intubated thoracoscopic surgery reduces the risk of transmission of infection in the perioperative period.

In conclusion, non-intubated anesthesia with a mask is feasible in patients scheduled for thoracic surgery. None of the patients in this series developed cough or vomiting during surgery. Unlike intubated general anesthesia and use of lung separation devices, non-intubated anesthesia avoids airway instrumentation. This may decrease the risk of contamination by airway secretions and conserve PPE.

## Data Availability Statement

The raw data supporting the conclusions of this article will be made available by the authors, without undue reservation.

## Ethics Statement

The studies involving human participants were reviewed and approved by Research Ethics Committee of National Taiwan University Hospital. Written informed consent for participation was not required for this study in accordance with the national legislation and the institutional requirements.

## Author Contributions

M-LW: conceptualization, methodology, investigation, and writing–original draft preparation. M-HH, H-HH, and Y-JC: supervision and validation. J-SC: conceptualization, funding acquisition, methodology, supervision, and writing–reviewing and editing. All authors contributed to the article and approved the submitted version.

## Funding

This work was supported in part by research grants from National Taiwan University Hospital (NTUH104-P08 to J-SC) and the Taiwan Lung Foundation (TLF2015-C02 to J-SC), Taipei, Taiwan. The funder had no role in the study design, data collection, analysis, decision to publish, or preparation of the manuscript.

## Conflict of Interest

The authors declare that the research was conducted in the absence of any commercial or financial relationships that could be construed as a potential conflict of interest.

## Publisher's Note

All claims expressed in this article are solely those of the authors and do not necessarily represent those of their affiliated organizations, or those of the publisher, the editors and the reviewers. Any product that may be evaluated in this article, or claim that may be made by its manufacturer, is not guaranteed or endorsed by the publisher.
